# Urine Osmolality Is a Potential Marker of Longer-Term Efficacy of Tolvaptan in Autosomal Dominant Polycystic Kidney Disease: A *Post Hoc* Analysis

**DOI:** 10.34067/KID.0000000000000485

**Published:** 2024-06-10

**Authors:** Vijay Ivaturi, Joga Gobburu, Bruce Leslie, Xiaofeng Wang, Pravin Jadhav

**Affiliations:** 1 Pumas-AI, Baltimore, Maryland; 2 Otsuka Pharmaceutical Development & Commercialization (OPDC), Inc., Princeton, New Jersey; 3 MDCI Biosciences LLC, Philadelphia, Pennsylvania

**Keywords:** ADPKD, CKD, clinical nephrology, clinical trial, GFR, interventional nephrology, osmolality, polycystic kidney disease, renal function decline

## Abstract

**Key Points:**

- *Post hoc* analyses of the TEMPO 3:4 trial suggest that short-term reductions in urine osmolality with tolvaptan predict effects on total kidney volume and eGFR.
- Change in urine osmolality has potential as a biomarker of treatment response and may facilitate trial design and clinical decision making.

**Background:**

Total kidney volume (TKV) and eGFR are measures of progression and treatment response in autosomal dominant polycystic kidney disease, but utility is limited by the long follow-up required for change assessment. In an analysis of data from the 3-year TEMPO 3:4 trial, we evaluated relationships among a short-term indicator of drug activity (change in urine osmolality [Uosm]) and longer-term outcomes to evaluate Uosm as a potential marker of efficacy.

**Methods:**

Linear regression modeling and single-point analyses assessed relationships among change in Uosm to week 3, change in TKV to month 12, and change in eGFR to month 36 in participants treated with tolvaptan (*n*=961) or placebo (*n*=483). Multivariate models evaluated the proportion of the tolvaptan treatment effect on eGFR attributable to change in Uosm.

**Results:**

Change in TKV to month 12 and Uosm to week 3 each correlated with change in eGFR to month 36, regardless of treatment assignment. A greater decrease in Uosm from baseline to week 3 was indicative of a slower decrease in eGFR to month 36 (slope estimate of −0.01, *P* < 0.00001). The effect of tolvaptan on Uosm accounted for 68.8% of the treatment effect on change in eGFR to month 36. Simulations of TEMPO 3:4 under the null hypothesis (*i.e*., replacement of all values for change in Uosm from baseline to week 3 with values from the placebo arm only) yielded a type 1 error rate indicating an acceptable risk of falsely concluding treatment efficacy on the basis of change in Uosm as a trial end point.

**Conclusions:**

Change in Uosm is a potential biomarker for long-term treatment outcome with tolvaptan and might expedite clinical trials and treatment decision making for drugs with similar mechanisms of action.

## Visual Abstract

**Figure:**
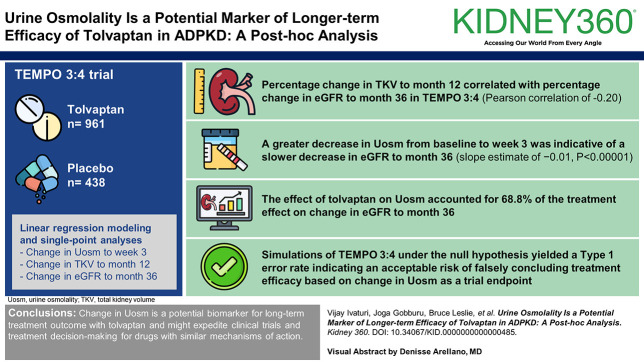

## Introduction

Although total kidney volume (TKV) and GFR are important outcomes for assessing disease progression and therapeutic benefit in autosomal dominant polycystic kidney disease (ADPKD), their utility for determining the response to treatment is limited, particularly in earlier stages of disease. Patients may have GFR in the normal range for decades as cyst burden increases and abnormalities in other kidney function parameters develop.^1–3^ Regular TKV assessment to evaluate treatment efficacy is not recommended, given variability in measurements over the short term.^4^ Before treatment, kidney volume measurement can be used to identify patients at risk of rapid disease progression before significant impairment of GFR has occurred.^5,6^ The US Food and Drug Administration and the European Medicines Agency have qualified TKV as a prognostic enrichment biomarker for selecting patients at high risk of kidney function decline for inclusion in clinical trials.^6^ The TKV-GFR relationship is consistent with ADPKD pathology, in which the expansion of cystic volume impinges on healthy tissue and destroys functioning nephrons.^7^

Tolvaptan inhibits cyst growth in ADPKD *via* competitive antagonism of vasopressin at the vasopressin type 2 receptor (V2R).^8^ The agent is indicated for slowing kidney function decline in patients with ADPKD who are at risk of rapid progression.^8^ The 3-year TEMPO 3:4 trial (NCT00428948) in adults with ADPKD demonstrated that slowing of TKV growth by tolvaptan over 12 months predicts reduction in the rate of eGFR decline over 36 months.^9^ The European Medicines Agency has accepted TKV as a surrogate clinical trial end point.^6^ In the context of regular clinical practice, however, such time frames are impractical, and shorter-term indicators for assessing response to therapy are needed.

Reduction in urine osmolality (Uosm) is a good marker of V2R antagonism because of the physiologic role of V2R signaling in promoting water reabsorption by the renal collecting ducts.^10^ The relationship of Uosm with the mechanism of action of tolvaptan, as well as ease of urine collection and Uosm measurement, support exploration of change in Uosm as an early indicator of therapeutic response to tolvaptan in ADPKD. Quantitative links between Uosm and subsequent rates of eGFR decline have previously been substantiated in analyses of TEMPO 3:4 trial data.^11^

The purpose of the *post hoc* analysis of TEMPO 3:4 reported here was to further evaluate relationships among short-term tolvaptan effects on Uosm and later changes in TKV and eGFR and thereby assess the utility of Uosm for decision making in therapeutics and drug development.

## Methods

Data were analyzed from TEMPO 3:4, a phase 3, randomized, double-blind, placebo-controlled, 3-year trial of tolvaptan in adults with ADPKD (961 randomized to and received tolvaptan, 484 randomized to placebo and 483 received placebo). By enrollment criteria, participants at baseline had largely intact kidney function (estimated creatinine clearance ≥60 ml/min) with a high likelihood of rapid disease progression (TKV ≥750 ml).

In TEMPO 3:4, nonfasting spot Uosm at trough concentration of study drug was determined, with sample collection immediately before morning dosing. According to the trial protocol, this sample was obtained from a second urine void taken after the first morning void and was ideally provided as a mid-stream, clean catch specimen. Assessments of TKV and eGFR were performed as reported.^9^

A series of *post hoc* analyses were performed to evaluate relationships among Uosm, TKV, and eGFR. Single-point analysis and linear regression modeling were conducted to explore the respective relationships between the following:

1. Percentage change in TKV from baseline to month 12 and percentage change in eGFR to month 36.
2. Change in Uosm from baseline to week 3 and percentage change in eGFR to month 36.

Owing to the known rapid-onset hemodynamic effect of tolvaptan, in which eGFR decreases after tolvaptan initiation and increases again after cessation of therapy,^12,13^ change in eGFR was measured from week 3 of tolvaptan treatment (end of titration) to end of treatment. This analysis of the chronic slope of eGFR excluded the acute hemodynamic effect. Only study participants with available Uosm, TKV, and eGFR data at baseline and the evaluated time points after treatment initiation were included in the analyses.

To assess the fraction of the tolvaptan treatment effect on eGFR that was attributable to the effect on Uosm, an analysis in three sequential steps was performed, consistent with earlier analyses conducted to establish the validity of surrogate end points.^14^ The steps were to (*1*) determine the magnitude of the treatment effect on change in eGFR to month 36 using a regression analysis with treatment as covariate; (*2*) test whether including change in Uosm to week 3 in the model modifies the treatment effect; and (*3*) compute the proportion of the treatment effect on change in eGFR to month 36 explained by change in Uosm to week 3 when treatment is removed from the model.

Finally, clinical trial simulations were conducted to ascertain that the false-positive rate of statistical inference is maintained at an acceptable level when using change in Uosm as a trial end point. The TEMPO 3:4 study was simulated, with all values for change in Uosm from baseline to week 3 resampled with replacement exclusively from the placebo participants to simulate under the null hypothesis. The regression equation was employed to calculate percentage change in eGFR to month 36. The residuals from the regression, which reflect the unexplained variability, were randomly added to the model prediction. The simulation process was repeated 2000 times, and the simulated placebo and tolvaptan arms were compared using a *t* test. The number of trials with significant difference between the treatment groups was counted.

## Results

In the TEMPO 3:4 trial, 740 of 961 participants randomized to tolvaptan (77%) and 417 of 484 participants randomized to placebo (86%) completed the 3-year study.^9^ For our analysis, participants were required to have evaluable data at baseline and the respective follow-up time point for each variable (Uosm, week 3; TKV, month 12; eGFR, month 36). As some participants were missing such data, the analysis sets were smaller than the 3-year completer populations. The analysis of the TKV–eGFR relationship included 732 participants from the tolvaptan arm and 415 participants from the placebo arm. The analysis of the Uosm–eGFR relationship included 685 participants from the tolvaptan arm and 397 participants from the placebo arm. Baseline characteristics of the analysis sets are shown in Supplemental Tables 1 and 2.

**Figure 1:**
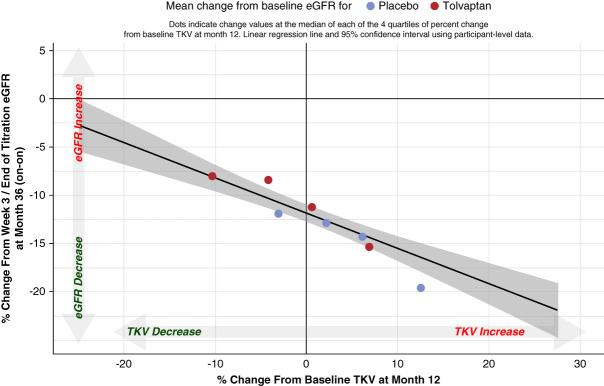
**Relationship between percent change in TKV from baseline to month 12 and percent change in eGFR from on-treatment baseline (week 3) to month 36 in TEMPO 3:4.** The quartiles are overlaid to represent trends in the raw data. The Pearson correlation for percent change in TKV to month 12 and percent change in eGFR to month 36 is −0.20. TKV, total kidney volume.

Percentage change in TKV to month 12 correlated with percentage change in eGFR to month 36 in TEMPO 3:4 (Pearson correlation of −0.20) (Figure 1). The TKV–eGFR relationship was independent of treatment arm, with similar slopes and intercepts for tolvaptan and placebo.

The robustness of the model was verified by calculating model-predicted values for mean percentage decline in eGFR at month 36 on the basis of the observed mean percentage TKV growth at month 12 in the placebo and tolvaptan arms. For the placebo arm, the model-predicted mean percentage decline in eGFR at month 36 was −13.71% (95% confidence interval, −14.70 to −12.72), and the observed value in TEMPO 3:4 was −14.69%. For the tolvaptan arm, the model-predicted mean percentage decline in eGFR at month 36 was −11.30% (95% confidence interval, −12.24 to −10.38), and the observed value in the trial was −10.75%. Thus, the model-predicted and observed values mapped closely.

Using similar methodology, the Uosm–TKV relationship was assessed, and as reported elsewhere,^15^ changes in Uosm at week 3 were indicative of changes in TKV at month 12 in the tolvaptan and placebo groups.

**Figure 2:**
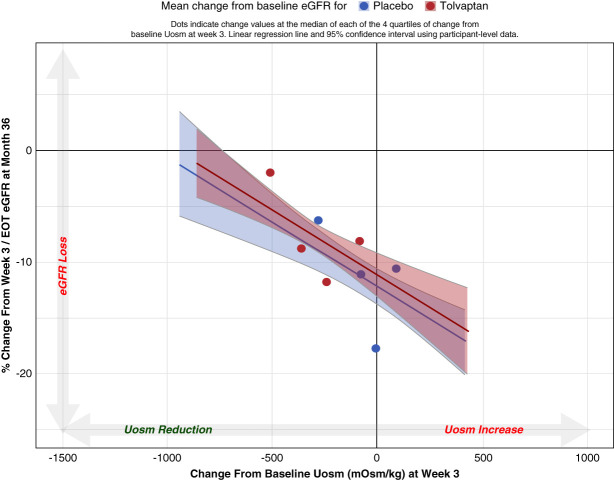
**Relationship between change in spot Uosm from baseline to week 3 and percent change in eGFR from on-treatment baseline (week 3) to month 36 in TEMPO 3:4.** The quartiles are overlaid to represent trends in the raw data. The Pearson correlation for change in Uosm to week 3 and percent change in eGFR to month 36 is 0.46. EOT, end of titration; Uosm, urine osmolality.

Next, the relationship of treatment-induced changes in spot Uosm at week 3 to treatment-induced percentage changes in eGFR at month 36 was evaluated. Greater decreases in spot Uosm at week 3 correlated with slower decrease in eGFR to month 36 (Pearson correlation of 0.46) (Figure 2). The slope estimate in the regression model was −0.01, *P* < 0.00001, indicating a 10% acceleration in change from baseline eGFR for every 1-unit increase in Uosm from baseline. The relationship was agnostic of trial treatment arm.

Multivariate modeling of patient-level data from TEMPO 3:4 using treatment as a covariate, after accounting for age and sex, demonstrated that tolvaptan exerted a significant effect on the change in eGFR to month 36 of 3.72% (*P* = 0.00018; Table 1). Inclusion in the model of Uosm change from baseline to week 3 modified the tolvaptan treatment effect, resulting in a decrease from 3.72% to 1.16% and loss of statistical significance (Table 2). Thus, the effect on Uosm accounted for 68.8% of the tolvaptan treatment effect on the change in eGFR to month 36 (100%×[1−1.16/3.72]).

**Table 1:** Results of a multivariate regression analysis of tolvaptan treatment effect on change in eGFR to month 36 in TEMPO 3:4

Parameter	Estimate	95% CI	*P* Value
Intercept, %	−10.35	−16.32 to −4.39	0.00068
Age, yr	−0.209	−0.343 to −0.074	0.00237
Sex (1, male; 2, female)	2.671	0.791 to 4.551	0.00539
**Model with treatment and the above predictors (0, placebo; 1, tolvaptan)**			
Treatment, %	3.72	1.78 to 5.66	0.00018

CI, confidence interval.

**Table 2:** Results of a multivariate regression analysis of tolvaptan treatment effect on change in eGFR to month 36 in TEMPO 3:4 with inclusion of change from baseline to week 3 in urine osmolality as a covariate

Parameter	Estimate	95% CI	*P* Value
Intercept, %	−12.91	−18.94 to −6.88	<0.00001
ΔUosm3, mOsm/kg	−0.01	−0.02 to −0.01	<0.00001
Age, yr	−0.169	−0.303 to −0.034	0.014
Sex (1, male; 2, female)	2.839	0.972 to 4.705	0.0029
**Model with treatment and the above predictors (0, placebo; 1, tolvaptan)**			
Treatment, %	1.160	−1.093 to 3.414	0.31

CI, confidence interval; ΔUosm3, change from baseline to week 3 in urine osmolality.

Simulations of TEMPO 3:4 trial were conducted to determine the false-positive rate of statistical inference when using change in Uosm as a trial end point, with all values for change in Uosm from baseline to week 3 resampled with replacement exclusively from the placebo participants. The 2000 simulations preserved the expected type 1 error (4.2% risk of a false-positive finding for change in eGFR to month 36 and 4.9% risk for change in Uosm to week 3), consistent with the typical threshold of acceptability (5%).

## Discussion

Results of the TEMPO 3:4 trial demonstrated that tolvaptan was associated with significant slowing of annual rates of increase in TKV and decline in kidney function relative to placebo in participants with ADPKD at elevated risk of rapid progression.^9^ Our *post hoc* analyses of data from TEMPO 3:4 suggest that short-term reductions in Uosm with tolvaptan are predictive of 12-month effects on TKV and 36-month effects on eGFR. The analyses also support a relationship between change in TKV to month 12 and change in eGFR to month 36. The latter finding was as expected, given that the most pronounced tolvaptan effects on TKV are seen within the first year of treatment.^16^

The linear relationship of short-term changes in Uosm and longer-term changes in eGFR as indicated by single-point analyses was substantiated by multivariate modeling that showed tolvaptan effects on Uosm accounting for 69% of the treatment effect on eGFR. Mechanisms other than vasopressin receptor inhibition may account for the additional effect. The results of TEMPO 3:4 trial simulations indicated that the type 1 error rate for demonstrating a significant difference between tolvaptan and placebo was 4.2% for an eGFR end point and 4.9% for a Uosm end point. Both type 1 error rates are consistent with the typical alpha of 5%, demonstrating the robustness of the Uosm-eGFR relationship.

The relationships of Uosm with effects on TKV and eGFR are supported by research on V2R physiology and the role of V2R activation in ADPKD.^10^ Antagonism of V2R by tolvaptan both counteracts the antidiuretic effect of vasopressin and inhibits the effects of vasopressin on cyst development and expansion.

It must be noted that while the 24-hour area under the curve of Uosm might be ideal to establish an empirical relationship with disease outcomes,^15^ such a measurement in large trials and especially in a clinical setting would be impractical. Furthermore, clinical trial simulations showed an acceptable type 1 error rate such that regulatory decision making would be similar using Uosm, TKV, or eGFR end points. Therefore, the spot Uosm (trough) measurement is still a reasonable trial end point to establish V2R antagonist efficacy.

The present analysis extends earlier research supporting Uosm as a potential marker of the effects of tolvaptan on kidney function decline.^11,15^ Health Canada has accepted the use of Uosm for guiding tolvaptan treatment. The Agency states that fasting morning Uosm provides the best guidance for dosing decisions to ensure target Uosm (*e.g*., ideally 300 mOsm/kg) for complete arginine vasopressin inhibition is achieved.^11^ Our study further demonstrates that short-term changes from baseline in Uosm can predict the long-term effects of tolvaptan on TKV and eGFR. Change in Uosm as a potential biomarker of treatment response might have utility for the goal of expediting and streamlining ADPKD clinical trials and possibly facilitate decision making by patients and health care providers.

## Supplementary Material

## Data Availability

Anonymized data created for the study are or will be available in a persistent repository on publication. Analyzable Data. Other. Otsuka repository. https://clinical-trials.otsuka.com.

## Supplemental Material

Supplemental Table 1. Baseline characteristics of TEMPO 3:4 participants included in the analysis of the relationship between percent change in TKV to month 12 and percent change in eGFR to month 36.

Supplemental Table 2. Baseline characteristics of TEMPO 3:4 participants included in the analysis of the relationship between change in Uosm to week 3 and percent change in eGFR to month 36.
