# Supplementary material for: Urine Osmolality Is a Potential Marker of Longer-Term Efficacy of Tolvaptan in Autosomal Dominant Polycystic Kidney Disease: A Post Hoc Analysis
Source: Kidney360. 2024 Jun 10;5(7):996–1001. doi: 10.34067/KID.0000000000000485 (PMC11296543; doi:10.34067/KID.0000000000000485)
Supplement: Supplementary file 1 [file kidney360-5-0996-s001.pdf]

ASN American Society of Nephrology
JASN Journal of the American Society of Nephrology
KIDNEY360 Accessing Our World From Every Angle
CJASN Clinical Journal of the American Society of Nephrology

ASN Journal Disclosure Form

As per ASN journal policy, I have disclosed any financial relationships or commitments I have held in the past 36 months as included below. I have listed my Current Employer below to indicate there is a relationship requiring disclosure. If no relationship exists, my Current Employer is not listed.

B. Leslie reports the following:
Employer: Otsuka Pharmaceutical Development and Commercialization; and Ownership Interest: Bristol-Myers Squibb, Pfizer.

I understand that the information above will be published within the journal article, if accepted, and that failure to comply and/or to accurately and completely report the potential financial conflicts of interest could lead to the following: 1) Prior to publication, article rejection, or 2) Post-publication, sanctions ranging from, but not limited to, issuing a correction, reporting the inaccurate information to the authors' institution, banning authors from submitting work to ASN journals for varying lengths of time, and/or retraction of the published work.

Name: Bruce R. Leslie
Manuscript ID: K360-2023-000168R3
Manuscript Title: Urine Osmolality Is a Potential Marker of Longer-term Efficacy of Tolvaptan in ADPKD
Date of Completion: May 27, 2024
Disclosure Updated Date: May 27, 2024

ASN
American Society of Nephrology
CJASN Kidney360 JASN

ASN Journal Disclosure Form

As per ASN journal policy, I have disclosed any financial relationship or commitment held by myself and/or my spouse/partner in the past 36 months as included below. I have listed my Current Employer below to indicate there is a relationship requiring disclosure. If no relationship exists, my Current Employer is not listed.

J. Gobburu reports the following:
Ownership Interest: Pumas-AI Inc.; Vivpro Corporation

I understand that the information above will be published within the journal article, if accepted, and that failure to comply and/or to accurately and completely report the potential financial conflicts of interest could lead to the following: 1) Prior to publication, article rejection, or 2) Post-publication, sanctions ranging from, but not limited to, issuing a correction, reporting the inaccurate information to the authors' institution, banning authors from submitting work to ASN journals for varying lengths of time, and/or retraction of the published work.

Name: Jogarao Gobburu
Manuscript ID: K360-2023-000168
Manuscript Title: Urine Osmolality Is a Marker of Longer-term Efficacy of Tolvaptan in ADPKD: Post-hoc Analysis of TEMPO 3:4
Date of Completion: March 3, 2023
Disclosure Updated Date: March 3, 2023

ASN American Society of Nephrology CJASN Kidney360 JASN

ASN Journal Disclosure Form

As per ASN journal policy, I have disclosed any financial relationship or commitment held by myself and/or my spouse/partner in the past 36 months as included below. I have listed my Current Employer below to indicate there is a relationship requiring disclosure. If no relationship exists, my Current Employer is not listed.

V. Ivaturi reports the following:
Employer: University of Maryland, Baltimore; Pumas-AI, Inc., CenterVille, VA; Consultancy: Otsuka US; Ownership Interest: Pumas-AI, Inc., CenterVille, VA; Patents or Royalties: Pumas-AI, Inc., CenterVille, VA; and Advisory or Leadership Role: Pumas-AI, Inc., CenterVille, VA.

I understand that the information above will be published within the journal article, if accepted, and that failure to comply and/or to accurately and completely report the potential financial conflicts of interest could lead to the following: 1) Prior to publication, article rejection, or 2) Post-publication, sanctions ranging from, but not limited to, issuing a correction, reporting the inaccurate information to the authors' institution, banning authors from submitting work to ASN journals for varying lengths of time, and/or retraction of the published work.

Name: Vijay Ivaturi
Manuscript ID: K360-2023-000168
Manuscript Title: Urine Osmolality Is a Marker of Longer-term Efficacy of Tolvaptan in ADPKD: Post-hoc Analysis of TEMPO 3:4
Date of Completion: March 4, 2023
Disclosure Updated Date: March 4, 2023

ASN
American Society of Nephrology
CJASN Kidney360 JASN

ASN Journal Disclosure Form

As per ASN journal policy, I have disclosed any financial relationship or commitment held by myself and/or my spouse/partner in the past 36 months as included below. I have listed my Current Employer below to indicate there is a relationship requiring disclosure. If no relationship exists, my Current Employer is not listed.

P. Jadhav reports the following:
Employer: Vivpro Corporation

I understand that the information above will be published within the journal article, if accepted, and that failure to comply and/or to accurately and completely report the potential financial conflicts of interest could lead to the following: 1) Prior to publication, article rejection, or 2) Post-publication, sanctions ranging from, but not limited to, issuing a correction, reporting the inaccurate information to the authors' institution, banning authors from submitting work to ASN journals for varying lengths of time, and/or retraction of the published work.

Name: Pravin Jadhav
Manuscript ID: K360-2023-000168
Manuscript Title: Urine Osmolality Is a Marker of Longer-term Efficacy of Tolvaptan in ADPKD: A Post-hoc Analysis
Date of Completion: April 5, 2023
Disclosure Updated Date: April 5, 2023

ASN
American Society of Nephrology
CJASN Kidney360 JASN

ASN Journal Disclosure Form

As per ASN journal policy, I have disclosed any financial relationship or commitment held by myself and/or my spouse/partner in the past 36 months as included below. I have listed my Current Employer below to indicate there is a relationship requiring disclosure. If no relationship exists, my Current Employer is not listed.

X. Wang reports the following:
Employer: Ostuka US

I understand that the information above will be published within the journal article, if accepted, and that failure to comply and/or to accurately and completely report the potential financial conflicts of interest could lead to the following: 1) Prior to publication, article rejection, or 2) Post-publication, sanctions ranging from, but not limited to, issuing a correction, reporting the inaccurate information to the authors' institution, banning authors from submitting work to ASN journals for varying lengths of time, and/or retraction of the published work.

Name: Xiaofeng Wang
Manuscript ID: K360-2023-000168
Manuscript Title: Urine Osmolality Is a Marker of Longer-term Efficacy of Tolvaptan in ADPKD: Post-hoc Analysis of TEMPO 3:4
Date of Completion: March 3, 2023
Disclosure Updated Date: March 3, 2023
