# Supplementary material for: Urine Osmolality Is a Potential Marker of Longer-Term Efficacy of Tolvaptan in Autosomal Dominant Polycystic Kidney Disease: A Post Hoc Analysis
Source: Kidney360. 2024 Jun 10;5(7):996–1001. doi: 10.34067/KID.0000000000000485 (PMC11296543; doi:10.34067/KID.0000000000000485)
Supplement: Supplementary file 2 [file kidney360-5-0996-s002.pdf]

Urine Osmolality Is a Potential Marker of Longer-term Efficacy of Tolvaptan in ADPKD: A Post-hoc Analysis

Vijay Ivaturi, Joga Gobburu, Bruce Leslie, Xiaofeng Wang, Pravin Jadhav

Supplemental Material

Supplemental Table 1. Baseline characteristics of TEMPO 3:4 participants included in the analysis of the relationship between percent change in TKV to month 12 and percent change in eGFR to month 36

Supplemental Table 2. Baseline characteristics of TEMPO 3:4 participants included in the analysis of the relationship between change in Uosm to week 3 and percent change in eGFR to month 36

Supplemental Table 1. Baseline characteristics of TEMPO 3:4 participants included in the analysis of the relationship between percent change in TKV to month 12 and percent change in eGFR to month 36

Characteristic	Tolvaptan (n=732)	Placebo (n=415)
Age in years, mean (SD)	39 (7)	39 (7)
Weight in kg, mean (SD)	79 (18)	78 (17)
Sex, n (%)		
Female	343 (47%)	198 (48%)
Male	389 (53%)	217 (52%)
Race, n (%)		
Asian	95 (13%)	58 (14%)
Black	11 (1.5%)	3 (0.7%)
White	616 (84%)	346 (83%)
Unknown	10 (1.4%)	8 (1.9%)
TKV in mL, mean (SD)	1695 (880)	1669 (870)
eGFR in mL/min/1.73 m^2, mean (SD)	81 (21)	82 (23)
EOT eGFR in mL/min/1.73 m^2, mean (SD)	76 (21)	81 (22)

eGFR, estimated glomerular filtration rate; EOT, end of titration; SD, standard deviation; TKV, total kidney volume.

Supplemental Table 2. Baseline characteristics of TEMPO 3:4 participants included in the analysis of the relationship between change in Uosm to week 3 and percent change in eGFR to month 36

Characteristic	Tolvaptan (n=685)	Placebo (n=397)
Age in years, mean (SD)	39 (7)	39 (7)
Weight in kg, mean (SD)	79 (18)	78 (17)
Sex, n (%)		
Female	318 (46%)	189 (48%)
Male	367 (54%)	208 (52%)
Race, n (%)		
Asian	89 (13%)	53 (13%)
Black	11 (1.6%)	3 (0.8%)
White	575 (84%)	332 (84%)
Unknown	10 (1.5%)	9 (2.3%)
Uosm in mOsm/kg, mean (SD)	489 (176)	513 (188)
eGFR in mL/min/1.73 m^2, mean (SD)	80 (21)	82 (23)
EOT eGFR in mL/min/1.73 m^2, mean (SD)	76 (21)	81 (22)

eGFR, estimated glomerular filtration rate; EOT, end of titration; SD, standard deviation; Uosm, urine osmolality.
